# Low-risk Lifestyle and Health Factors and Risk of Mortality and Vascular Complications in Chinese Patients With Diabetes

**DOI:** 10.1210/clinem/dgac264

**Published:** 2022-04-23

**Authors:** Zhijia Sun, Yizhen Hu, Canqing Yu, Yu Guo, Yuanjie Pang, Dianjianyi Sun, Pei Pei, Ling Yang, Yiping Chen, Huaidong Du, Jianrong Jin, Sushila Burgess, Alex Hacker, Junshi Chen, Zhengming Chen, Jun Lv, Liming Li

**Affiliations:** Department of Epidemiology & Biostatistics, School of Public Health, Peking University, Beijing 100191, China; Department of Epidemiology & Biostatistics, School of Public Health, Peking University, Beijing 100191, China; Department of Epidemiology & Biostatistics, School of Public Health, Peking University, Beijing 100191, China; Peking University Center for Public Health and Epidemic Preparedness & Response, Beijing 100191, China; Fuwai Hospital Chinese Academy of Medical Sciences, Beijing 100037, China; Department of Epidemiology & Biostatistics, School of Public Health, Peking University, Beijing 100191, China; Department of Epidemiology & Biostatistics, School of Public Health, Peking University, Beijing 100191, China; Chinese Academy of Medical Sciences, Beijing 100730, China; Medical Research Council Population Health Research Unit at the University of Oxford, Oxford OX3 7LF, United Kingdom; Clinical Trial Service Unit & Epidemiological Studies Unit (CTSU), Nuffield Department of Population Health, University of Oxford, Oxford OX3 7LF, United Kingdom; Medical Research Council Population Health Research Unit at the University of Oxford, Oxford OX3 7LF, United Kingdom; Clinical Trial Service Unit & Epidemiological Studies Unit (CTSU), Nuffield Department of Population Health, University of Oxford, Oxford OX3 7LF, United Kingdom; Medical Research Council Population Health Research Unit at the University of Oxford, Oxford OX3 7LF, United Kingdom; Clinical Trial Service Unit & Epidemiological Studies Unit (CTSU), Nuffield Department of Population Health, University of Oxford, Oxford OX3 7LF, United Kingdom; Wuzhong CDC, Jiangsu 215128, China; Clinical Trial Service Unit & Epidemiological Studies Unit (CTSU), Nuffield Department of Population Health, University of Oxford, Oxford OX3 7LF, United Kingdom; Clinical Trial Service Unit & Epidemiological Studies Unit (CTSU), Nuffield Department of Population Health, University of Oxford, Oxford OX3 7LF, United Kingdom; China National Center for Food Safety Risk Assessment, Beijing 100022, China; Clinical Trial Service Unit & Epidemiological Studies Unit (CTSU), Nuffield Department of Population Health, University of Oxford, Oxford OX3 7LF, United Kingdom; Department of Epidemiology & Biostatistics, School of Public Health, Peking University, Beijing 100191, China; Peking University Center for Public Health and Epidemic Preparedness & Response, Beijing 100191, China; Key Laboratory of Molecular Cardiovascular Sciences (Peking University), Ministry of Education, Beijing 100191, China; Department of Epidemiology & Biostatistics, School of Public Health, Peking University, Beijing 100191, China; Peking University Center for Public Health and Epidemic Preparedness & Response, Beijing 100191, China

**Keywords:** risk factors, lifestyle, diabetes mellitus, diabetes complications, mortality, cohort study

## Abstract

**Background:**

There is an evidence gap about whether a low-risk lifestyle is as important as achieving blood pressure (BP) and random blood glucose (RBG) control.

**Objectives:**

To explore the long-term impacts and relative importance of low-risk lifestyle and health factors on the risk of all-cause and cancer mortality and macrovascular and microvascular complications among patients with diabetes.

**Methods:**

This study included 26,004 diabetes patients in the China Kadoorie Biobank. We defined 5 lifestyle factors (smoking, alcohol drinking, physical activity, fruit and vegetable intake, and waist-to-hip ratio) and 2 health factors (BP and RBG). Cox regression was used to yield adjusted hazard ratios (HRs) and CIs for individual and combined lifestyle and health factors with the risks of diabetes-related outcomes.

**Results:**

There were 5063 deaths, 6848 macrovascular complications, and 2055 microvascular complications that occurred during a median follow-up of 10.2 years. Combined low-risk lifestyle factors were associated with lower risk of all main outcomes, with HRs (95% CIs) for participants having 4 to 5 low-risk factors vs 0 to 1 of 0.50 (0.44-0.57) for all-cause mortality, 0.55 (0.43-0.71) for cancer mortality, 0.60 (0.54-0.67) for macrovascular complications, and 0.75 (0.62-0.91) for microvascular complications. The combined 4 to 5 low-risk lifestyle factors showed relative importance in predicting all-cause and cancer mortality and macrovascular complications.

**Conclusions:**

Assuming causality exists, our findings suggest that adopting a low-risk lifestyle should be regarded as important as achieving ideal BP and glycemic goals in the prevention and management of diabetes-related adverse outcomes.

Diabetes has become a major global health issue. In 2019, the global diabetes prevalence of adults aged 20 to 79 years was 9.3%, and China had the largest number of diabetic patients (1). Diabetes is associated with an increased death risk from all causes, cancer, and cardiovascular disease (CVD) (2). Also, macrovascular and microvascular complications are responsible for much of the diabetes-related burden (3). These findings highlight the importance of the prevention and management of diabetes-related adverse outcomes.

Evidence from Western and Chinese populations supports that adhering to a healthy lifestyle reduces the risk of newly developing type 2 diabetes (4, 5). However, studies focusing on the long-term impacts of combined lifestyle factors on adverse outcomes in diabetic patients are limited. A recent meta-analysis summarized 10 prospective studies, mostly from high-income countries and Western populations. The pooled results showed that adopting a healthy lifestyle was associated with a lower risk of all-cause, CVD, and cancer mortality and incident CVD among diabetic patients (4). However, prospective evidence specific for lifestyle and microvascular complications is lacking.

Quite a few research studies have focused on glycemic, blood pressure (BP), and lipid control and their prognosis for diabetic patients. However, limited prospective studies have examined the role of multiple lifestyle and metabolic measures simultaneously in preventing cardiovascular complications from diabetes (6). To prevent adverse outcomes in diabetic patients, whether low-risk lifestyle yields equivalent outcomes compared with achieving BP and random blood glucose (RBG) control, there is still an evidence gap to be filled. Only 1 study of the Swedish population compared the relative importance of socioeconomic, lifestyle, and health factors on all-cause mortality and CVD outcomes for diabetic patients (7). More studies are needed, especially in low- and middle-income countries with limited health care resources.

Using data from the China Kadoorie Biobank (CKB) study, we explored the long-term impacts of both low-risk lifestyle and health factors on the risk of all-cause and cancer mortality and macrovascular and microvascular complications among diabetic patients. We further compared the relative importance of adopting a healthy lifestyle vs BP and glycemic control on preventing long-term diabetes-related problems.

## Materials and Methods

### Study Population

The CKB study is a large nationwide prospective cohort study with an enrollment of 512,725 participants aged 30 to 79 years. From 2004 to 2008, the baseline survey took place in 5 urban and 5 rural localities across China to provide diversity in geographical and social characteristics. About 5% of randomly chosen participants were included in the resurvey every 5 years. One of the resurveys took place in 2013 and 2014, which contained 25,041 participants. Details of the study design, methods, and population characteristics have been reported previously (8, 9). The CKB study received approval from the Ethical Review Committee of the Chinese Centre for Disease Control and Prevention and the Oxford Tropical Research Ethics Committee, University of Oxford. All participants provided written informed consent.

In the present analysis, we included 30,300 diabetic participants at baseline, defined as either having a self-reported clinician diagnosis of diabetes or having screen-detected diabetes. Screen-detected diabetes was defined as (1) RBG ≥ 7.0 mmol/L with time since last meal of ≥ 8 hours or RBG ≥ 11.1 mmol/L with time since last meal of < 8 hours or (2) a fasting blood glucose level ≥ 7.0 mmol/L on subsequent testing among participants whose onsite RBG level between 7.8 and 11.0 mmol/L (10). Additionally, we excluded participants with self-reported clinician diagnosis of heart disease (n = 2,699), stroke (n = 1,537), or cancer (n = 294), and those who had missing data for RBG (n = 158), leaving 26,004 participants for the current analysis.

### Definition of Low-risk Factors

Details of the baseline data collection are introduced in the Supplementary Methods (11). We considered 7 factors in the current study, including 5 lifestyle factors (smoking, alcohol drinking, physical activity, dietary habits, and waist-to-hip ratio [WHR]) based on the previous studies and 2 health factors (BP and RBG) (4). The adiposity measure of WHR was considered as a surrogate of balance between energy intake and expenditure (12). Blood lipids were not included in the study because we did not tested them in all participants.

The low-risk group for smoking included nonsmokers or those who had stopped smoking for reasons other than illness for at least 6 months. The smokers who had quit for illness were combined with current regular smokers to avoid bias. The low-risk group for alcohol drinking was those never-regular drinkers, weekly drinkers, or daily drinkers with an intake of < 30 g of pure alcohol for men or < 15 g for women. Similar to the definition for smoking, former alcohol drinkers were excluded from the low-risk group. For physical activity, the low-risk group was defined as engaging in a sex-specific median or higher level of physical activity. Eating both fruits and vegetables daily was considered a low-risk dietary habit. Participants with a WHR < 0.90 in men and < 0.85 in women constituted the low-risk group.

We defined the cutoff values of BP and RBG based on guideline-recommended target levels for patients with type 2 diabetes (13-15). The low-risk group for BP was considered having measured systolic blood pressure (SBP) < 130 mmHg and diastolic blood pressure < 80 mmHg, regardless of whether receiving antihypertensive therapy. The low-risk group for RBG was defined as RBG < 10.0 mmol/L with fasting for < 8 hours or RBG < 7.0 mmol/L with fasting for ≥ 8 hours.

The numbers of the low-risk lifestyle factors (range, 0-5) and health factors (range, 0-2) were counted separately or combined (range, 0-7).

### Ascertainment of Outcomes

Mortality and morbidity since the participants’ enrollment into the study were identified through linkages with death and disease registries and health insurance databases and active follow-up. The International Statistical Classification of Diseases and Related Health Problems, 10th revision, was used to code all cases by trained staff who did not know the baseline information.

The primary study outcomes were all-cause mortality, cancer mortality, and diabetes-related macrovascular and microvascular complications. Macrovascular complications comprised the total cardiovascular mortality and incidence of stroke and myocardial infarction. Microvascular complications included nephropathy, retinopathy, and neuropathy. We also separately analyzed major coronary events ([MCEs] including ischemic heart disease mortality and myocardial infarction incidence), ischemic stroke, hemorrhagic stroke (HS), and each microvascular complication as secondary outcomes. The International Statistical Classification of Diseases and Related Health Problems, 10th revision, codes of diseases are described in Table S1 (11).

### Statistical Analysis

The mean values and proportions for baseline characteristics of participants by baseline diabetes status and the number of low-risk lifestyle factors were adjusted for age (years), sex, and study area, where appropriate. Person-years at risk were calculated from the date of the baseline survey to the date of outcomes diagnosis, death, loss to follow-up, or December 31, 2017, whichever came first.

We constructed Cox proportional hazards models to calculate hazard ratios (HRs) and 95% CIs for the associations of individual risk factors or the number of combined risk factors with the risks of each outcome. Models were stratified jointly by study area and age at baseline in 5-year intervals and adjusted for age, sex, education, body mass index at baseline, family history of diabetes, family histories of heart attack, stroke, or cancer, diabetes duration, diabetic treatment, statin use, and aspirin use. Diabetes duration was defined as 0 years for screen-detected diabetes participants.

When analyzing individual low-risk factors, all other low-risk factors were included in the model simultaneously. We first examined the full sample and then repeated the analysis separately for self-reported and screen-detected diabetes patients. For analysis of the number of lifestyle factors, the model was further adjusted for SBP and RBG level at baseline. For analysis of the number of health factors, the model was further adjusted for smoking status, alcohol consumption, intake frequency of fresh fruits and vegetables, physical activity, and WHR. The linear trend test for combined low-risk factors was performed by treating the number of low-risk factors as a continuous variable.

Several sensitivity analyses were performed to test the robustness of the results: (1) excluding cases identified during the first 2 years of follow-up; (2) further adjusting for prevalent hypertension (yes or no); (3) defining low risk of BP with different cutoff values according to previous studies; (4) excluding hypertensive patients who had measured SBP < 130 mmHg and DBP <  80 mmHg at baseline from the low-risk group; and (5) applying a Bonferroni correction to the analysis of individual low-risk factors. We divided 0.05 by the number of primary outcomes and estimated 98.75% (1-0.05/4) CIs accordingly.

We assessed the relative importance of single lifestyle and health factors and combined lifestyle factors in terms of predicting the outcomes using the Cox models formulated previously. Explainable log-likelihood attributable to each low-risk factor (ie, the proportion of Wald χ ^2^ statistics) quantified the relative importance of each factor (7, 16). The relative strength of the combined lifestyle factor was assessed by comparing participants with 4 to 5 low-risk lifestyle factors with those with 0 to 1 health lifestyle factor. We further stratified the analysis by sex, age at baseline, and region. Computations were performed using rms package of R software (17).

All *P* values were 2-sided, and statistical significance was defined as *P* <  0.05. Statistical analyses were conducted with Stata (version 15.0, StataCorp) and R 4.1.0.

## Results

### Study Population

Of the 26,004 diabetes patients included in the analysis, 13,027 (50.1%) were previously diagnosed with diabetes, and 12,977 (49.9%) had screen-detected diabetes at baseline (Table 1). The mean age of the participants was 57.4 years (SD 9.6), 61.2% were women, and 58.6% were urban residents. Of all included diabetes patients, 14.4% adopted at least 4 low-risk lifestyle factors at baseline; 26.4% and 23.2% kept BP or RBG in target ranges, respectively. Only a few participants took statin (0.5%) or aspirin (1.4%). Most participants did not change their lifestyle between baseline survey and resurvey in 2013 and 2014 (Table S2 (11)).

**Table 1.** Baseline characteristics of participants according to baseline diabetes status and number of low-risk lifestyle factors

Baseline characteristics	Screen-detected diabetes (*n* =12,977)					Self-reported diabetes (*n* =13,027)				
	0	1	2	3	≥4	0	1	2	3	≥4
No. of participants, n (%)	301	1284	4253	5087	2052	324	1356	4922	4727	1698
	(2.3)	(9.9)	(32.8)	(39.2)	(15.8)	(2.5)	(10.4)	(37.8)	(36.3)	(13.0)
Female, %	2.0	10.6	54.6	74.8	77.3	4.0	12.8	62.7	73.8	75.9
Age, year (SD)	59.4	57.5	57.9	55.1	53.0	60.5	58.9	60.4	57.5	56.4
	(9.6)	(10.0)	(9.9)	(9.4)	(9.9)	(8.7)	(9.4)	(8.9)	(9.0)	(9.1)
Urban area, %	58.1	57.9	53.4	49.7	68.7	59.9	63.3	60.9	58.8	75.8
Middle school and higher, %	35.2	39.8	41.6	45.2	52.5	40.2	42.2	44.2	49.2	56.5
Body mass index, kg/m^2^ (SD)	25.8	25.9	25.7	25.2	23.5	25.6	25.3	25.1	24.6	23.3
	(3.2)	(3.4)	(3.7)	(3.7)	(3.7)	(3.5)	(3.3)	(3.4)	(3.5)	(3.4)
Family history, %										
Diabetes	9.4	13.9	11.1	12.4	13.0	25.9	23.3	22.5	23.4	25.0
Heart attack	3.4	3.6	3.2	3.4	3.5	2.7	3.9	3.7	4.2	4.3
Stroke	19.7	19.1	19.0	19.6	18.5	21.6	24.1	20.4	21.5	21.1
Cancer	20.0	18.3	16.1	17.6	17.1	21.4	18.7	18.6	19.6	18.6
Having low-risk lifestyle factors, %										
Non-current smoking*	--	32.2	68.4	83.7	95.7	--	33.8	73.6	87.5	96.3
Non-excessive alcohol drinking^†^	--	69.1	88.6	97.0	99.5	--	73.5	91.7	97.2	99.0
Eating fruits and vegetables daily	--	3.2	6.5	24.7	54.7	--	4.0	6.0	24.0	53.7
Actively engaging in physical activity^‡^	--	23.2	30.6	72.3	89.9	--	16.5	20.6	64.5	89.1
WHR<0.90 (men) or 0.85 (women)	--	4.5	10.4	24.1	72.1	--	5.2	8.9	29.0	71.1
Diabetes status										
Random blood glucose, mmol/L (SD)	13.2	13.4	13.3	13.1	12.6	11.7	11.9	11.7	11.3	10.8
	(4.8)	(5.3)	(5.4)	(5.4)	(5.2)	(6.0)	(6.0)	(5.9)	(5.7)	(5.5)
Receiving diabetic treatment^§^, %	--	--	--	--	--	86.9	84.7	86.0	83.4	81.0
Diabetes duration, year (SD)	--	--	--	--	--	5.9 (4.9)	5.6 (4.8)	5.8 (5.1)	6.0 (5.2)	6.2 (5.4)
Hypertension status										
SBP, mmHg (SD)	144.0	143.5	142.7	141.3	137.6	143. 1	142. 3	142. 2	140. 2	137. 9
	(23.1)	(21.9)	(22.8)	(22.5)	(22.0)	(21.5)	(22.0)	(22.8)	(22.1)	21.2)
DBP, mmHg (SD)	82.4	82.1	81.7	81.0	78.5	79.6	79.7	79.4	78.5	77.5
	(11.7)	(11.9)	(11.6)	(11.2)	(11.0)	(12.4)	(11.2)	(11.1)	(10.7)	(10.4)
Prevalent hypertension, %	58.7	60.6	59.2	55.6	47.5	62.5	64.0	62.2	57.4	53.1
Receiving hypertension treatment^||^,%	21.2	22.5	21.3	19.9	14.9	31.6	31.4	29.3	26.0	24.1
Taking statin,%	0.3	0.2	0.3	0.4	0.2	0.8	0.7	0.7	0.9	1.0
Taking aspirin,%	1.2	1.4	1.2	0.8	0.7	1.0	2.3	1.9	2.4	1.7

Values are means (SD) or percentages with adjustment for age (years), sex, and study area, where appropriate.

Abbreviations: DBP, diastolic blood pressure; SBP, systolic blood pressure; WHR, waist-to-hip ratio.

^*a*^Including never smokers and participants who had stopped smoking for reasons other than illness.

^*b*^Including never alcohol drinkers and participants who drank < 30 g/d of pure alcohol in men or < 15 g/d in women. Former drinkers were excluded.

^*c*^Engaging in sex-specific median or higher level of physical activity.

^*d*^Taking insulin and/or oral hypoglycemic drugs.

^*e*^Taking angiotensin-converting enzyme inhibitors, β-blockers, diuretics, or calcium antagonists.

During a median of 10.2 years (a total of 265,700 person-years) of follow-up, we documented 5063 deaths, 2091 because of cardiovascular disease, and 1133 because of cancer. We also documented 6848 macrovascular complications (MCE: 1314; IS: 5092; HS: 896) and 2055 microvascular complications (nephropathy: 771; retinopathy: 781; neuropathy: 888).

### Individual Low-risk Factors

All lifestyle and health factors were associated with all-cause mortality, and most were associated with macrovascular and microvascular complications (Table S3 (11)). Participants who were defined as being at low risk of smoking and physical activity showed reduced risks of all main outcomes (Table 2). The other 3 low-risk lifestyle factors were also associated with lower risks of some of the study outcomes. The ideal levels for BP and RBG were consistently associated with lower risks of all-cause mortality and macrovascular and microvascular complications (Table 2). Associations were generally similar for both screen-detected and self-reported diabetes patients (Table S4 (11)). After Bonferroni correction, some marginal associations were no longer statistically significant, such as the association between WHR and all-cause mortality (Table S5 (11)).

**Table 2.** Adjusted hazard ratios (95% CIs) for mortality and diabetes complications

	All-cause mortality			Cancer mortality			Macrovascular complications			Microvascular complications		
	Deaths	Deaths /PYs (/1,000)	HRs (95% CIs)	Deaths	Deaths /PYs (/1,000)	HRs (95% CIs)	Cases	Cases /PYs (/1,000)	HRs (95% CIs)	Cases	Cases /PYs (/1,000)	HRs (95% CIs)
Lifestyle factors												
Non-current smoking	3351	17.0	0.79 (0.73-0.85)	720	3.7	0.79 (0.67-0.93)	4908	27.3	0.84 (0.79-0.90)	1500	7.9	0.87 (0.76-0.99)
Non-excessive alcohol drinking	4269	18.2	0.86 (0.78-0.93)	910	3.9	0.74 (0.62-0.88)	6002	28.0	0.94 (0.86-1.02)	1819	8.0	1.08 (0.93-1.26)
Eating fruits and vegetables daily	750	14.1	0.82 (0.75-0.89)	228	4.3	0.97 (0.83-1.14)	1307	27.1	0.86 (0.80-0.91)	365	7.1	0.92 (0.81-1.04)
Actively engaging in physical activity	1734	12.6	0.71 (0.67-0.76)	433	3.1	0.83 (0.72-0.95)	2621	20.4	0.87 (0.83-0.92)	906	6.8	0.89 (0.81-0.99)
WHR<0.90 (men) or 0.85 (women)	1179	19.9	0.91 (0.85-0.99)	275	4.6	0.97 (0.83-1.13)	1329	24.2	0.85 (0.79-0.91)	429	7.5	0.93 (0.82-1.04)
Health factors												
Blood pressure<130/80 mmHg	1016	14.2	0.80 (0.75-0.86)	282	4.0	1.09 (0.95-1.26)	1296	19.4	0.69 (0.64-0.73)	520	7.5	0.89 (0.81-0.99)
Random blood glucose<10.0/7.0mmol/L	948	15.1	0.65 (0.61-0.70)	258	4.1	0.92 (0.79-1.06)	1571	27.6	0.82 (0.77-0.87)	385	6.3	0.57 (0.51-0.64)

The multivariable model was adjusted for age (years), sex, education (no formal school, primary school, middle school, high school, college/university or higher), body mass index (kg/m^2^), family history of diabetes (yes or no), family histories of heart attack and stroke (yes or no, only in the analyses of all-cause mortality and macrovascular complications), family history of cancer (yes or no, only in the analyses of all-cause and cancer mortality), diabetes duration (years), diabetic treatment (yes or no), statin use (yes or no), and aspirin use (yes or no). All lifestyle and health factors were included simultaneously in the same model.

Low-risk lifestyle factors were defined as: never smoking or having stopped for reasons other than illness; never drinking or current drinking < 30 g/d of pure alcohol in men or < 15 g/d in women (former drinkers not included); eating fruits and vegetables every day; engaging in a sex-specific median or higher level of physical activity; and having a WHR < 0.90 in men and < 0.85 in women.

Low-risk health factors were defined as: systolic blood pressure < 130 mmHg and diastolic blood pressure < 80 mmHg; and random blood glucose < 10.0 mmol/L (fasting for < 8 hours) or < 7.0 mmol/L (fasting for ≥ 8 hours).

Abbreviations: HR, hazard ratio; PY, person-year; WHR, waist-to-hip ratio.

In the analyses of secondary outcomes, low-risk lifestyle factors were mainly associated with reduced risks of each macrovascular complication (Tables S6 and S7 (11)). However, both low-risk health factors were associated with almost all macrovascular and microvascular complications.

### Combined Low-risk Factors

**Figure 1.**
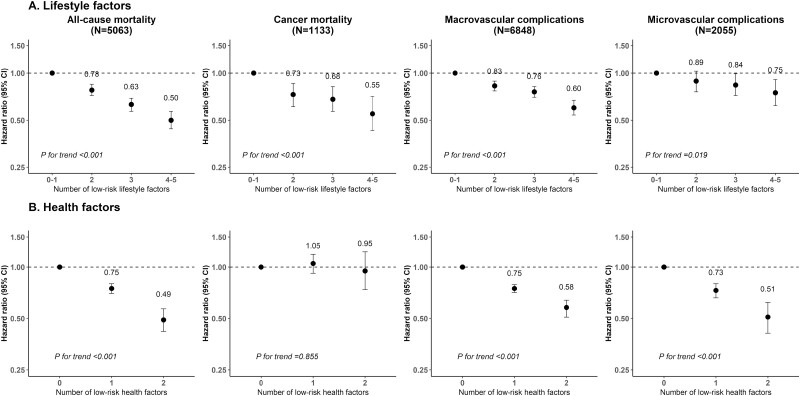
Adjusted hazard ratios (95% CIs) for mortality and diabetes complications by the number of low-risk lifestyle and health factors. Please refer to Table 2 for the definitions of low-risk factors and covariates adjusted in the models. The analyses of lifestyle factors were further adjusted for systolic blood pressure (mmHg) and random blood glucose (mmHg). The analyses of health factors were further adjusted for tobacco smoking (nonsmokers, former smokers who quit smoking for a nonillness reason, current smokers and former smokers who quit smoking because of illness: 1-14, 15-24, or ≥ 25 cigarettes or equivalent per day), alcohol consumption (never drinkers, former drinkers, current drinkers: less than daily or drinking < 30 g/d of pure alcohol in men or < 15 g/d in women, drinking ≥ 30 g/d of pure alcohol in men or ≥ 15 g/d in women), intake frequency of fresh fruits and vegetables (days/wk: calculated by assigning participants to the midpoint of their consumption category), physical activity (MET-hours/d), and waist-hip ratio.

The risks of all primary outcomes gradually decreased with the increase in the number of low-risk lifestyle factors (all *P*_trend_ < 0.05) (Fig. 1A and Table S8 (11)). Compared with participants with 0 to 1 low-risk lifestyle factors, those with 4 or 5 low-risk lifestyle factors had an HR (95% CI) of 0.50 (0.44-0.57) for all-cause mortality, 0.55 (0.43-0.71) for cancer mortality, 0.60 (0.54-0.67) for macrovascular complications, and 0.75 (0.62-0.91) for microvascular complications. Similar trends were also observed for MCE, ischemic stroke, and diabetic nephropathy.

**Figure 2.**
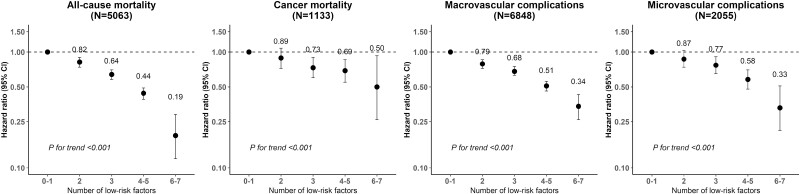
Adjusted hazard ratios (95% CIs) for mortality and diabetes complications by the number of low-risk factors. Please refer to Table 2 for the definitions of low-risk factors and covariates adjusted in the models.

The number of low-risk health factors was also inversely associated with the risk of all-cause mortality and macrovascular and microvascular complications in combined or individual outcomes (all *P*_trend_ < 0.05), but not with cancer mortality (Fig. 1B and Table S9 (11)). The corresponding HR (95% CI) for participants with both BP and RBG at the ideal level, compared with those with neither, was 0.49 (0.42-0.57), 0.58 (0.52-0.64), and 0.51 (0.42-0.62) for all-cause mortality, macrovascular complications, and microvascular complications, respectively. When 7 low-risk lifestyle and health factors were considered together, all primary and secondary outcomes, except the diabetic neuropathy, were inversely associated with the number of low-risk factors (all *P*_trend_ < 0.001) (Fig. 2 and Table S10 (11)).

The results were not substantially altered after excluding cases identified during the first 2 years of follow-up or further adjustment for prevalent hypertension (Tables S11 and S12 (11)). The changes in the cutoff for BP and the exclusion of hypertensive patients from the low-risk group did not change our findings qualitatively (Table S13 (11)).

### Relative Importance of Low-risk Factors in Predicting the Outcomes

**Figure 3.**
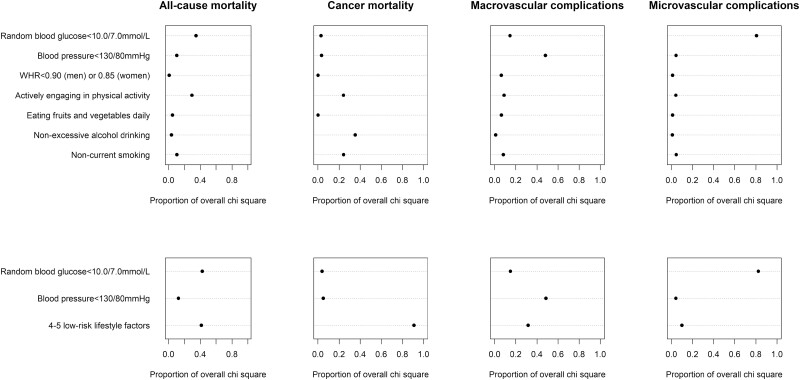
Relative importance of lifestyle and health factors for risks of mortality and diabetes complications. The relative importance of each low-risk factors was measured by estimating explained log-likelihood, with larger proportion of overall χ ^2^ indicating greater importance. Please refer to Table 2 for the definitions of low-risk factors and covariates adjusted in the models.

The 2 strongest predictors regarding the risk of all-cause mortality were RBG and physical activity (Fig. 3). The 3 strongest predictors for cancer mortality were alcohol drinking, smoking, and physical activity. The strongest predictor for macrovascular and microvascular complications was BP and RBG, respectively.

The combination of 4 or 5 low-risk lifestyle factors kept showing their relative importance in predicting all-cause and cancer mortality and macrovascular complications. By contrast, the RBG was important for all-cause mortality and microvascular complications and BP for macrovascular complications. Further stratification analyses showed broadly consistent findings among male participants, those aged 50 years and older, and urban and rural residents with those among the entire population. However, the combined low-risk lifestyle had diminished importance in predicting all-cause and cancer mortality for females and predicting macrovascular complications for participants aged younger than 50 years (Figs. S1, S2, and S3).

## Discussion

In this Chinese diabetes population, fewer than one-sixth adhered to 4 or 5 low-risk lifestyle factors, and one-fourth reached the ideal BP and RBG level. The combined low-risk lifestyle factors were associated with a lower risk of all-cause and cancer mortality and macrovascular and microvascular complications. A similar inverse association was also observed for well-controlled BP and RBG and risk of all-cause mortality and macrovascular and microvascular complications. Compared with diabetic patients with 0 to 1 low-risk lifestyle and health factors, those with 6 to 7 had a reduced risk of all-cause and cancer mortality and macrovascular and microvascular complications by 81%, 50%, 66%, and 67%, respectively. Our findings showed the importance of adopting a healthy lifestyle in preventing all-cause and cancer mortality and macrovascular complications.

A recent meta-analysis summarized the findings of combined lifestyle factors on the prognosis of 34,385 type 2 diabetic patients (4). Of the 10 studies included, sample sizes ranging from 592 to 11,527 and follow-up time ranging from 4.0 to 20.6 years, only 2 were Asian studies. The pooled HR of 7 studies was 0.44 (95% CI, 0.33-0.60) for all-cause mortality when comparing participants of the least-healthy lifestyle with those of the healthiest lifestyle. Only 3 studies from Western populations had composite CVD events as the outcome (18-20) and achieved a pooled HR of 0.48 (0.37-0.63) (4). Our study further added to the evidence that combined low-risk lifestyle factors had the strongest impact on the risk of MCE, followed by ischemic stroke and HS. Another study using data from CKB explored the impacts of lifestyle factors on the progression of cardiometabolic multimorbidity using the multistate model (21). In this study, lifestyle factors were assessed when participants were without diabetes or CVDs, and incident cases of type 2 diabetes were identified during follow-up. The findings of both studies complement each other, suggesting that adopting a healthy lifestyle before and after developing diabetes is both important in preventing diabetic cardiovascular complications and death. Three studies with cancer mortality as an outcome were all underpowered because of the small sample size, ranging from 2125 to 5686 (22-24). Their pooled HR was 0.69 (95% CI, 0.47-1.00) (4), which was consistent with our findings that a low-risk lifestyle was associated with reduced risk of cancer mortality for diabetic patients.

Our analysis showed that a low-risk lifestyle was associated with a lower risk of microvascular complications, especially diabetic nephropathy. The 2776 surviving participants of the Diabetes Prevention Program (1996-2001), a randomized controlled trial, were followed for an average of 15 years after the trial (25). The prevalence of microvascular complications at the end of follow-up was lower in the original lifestyle intervention arm (8.7%) than the placebo and metformin arms (11% and 11.2%) in women; but there was no difference among 3 arms in the total population. The 576 adults with impaired glucose tolerance of Da Qing Diabetes Prevention Study (1986-1992) were followed for up to 30 years after the trial (26). The incidence of microvascular complications was 35% (95% CI, 5-55) lower in the original intervention groups than the control group.

With regard to the impacts of the ideal level of BP or glycemia, our findings were consistent with previous evidence. Pooled results of 40 trials with 100,354 participants showed that BP-lowering treatment was associated with lower risks of all-cause mortality, CVD events, retinopathy, and albuminuria in diabetic patients (27). In 3642 diabetic patients from the UK Prospective Diabetes Study, per 1% reduction in updated mean glycated hemoglobin (HbA1c) was associated with 37%, 14%, 12%, and 14% decrease in risk for microvascular complications, myocardial infarction, stroke, and all-cause mortality after a median 10 years of follow-up, respectively (28). Similar to our findings, the results of UK Prospective Diabetes Study showed that the increase in the incidence rates for microvascular disease with hyperglycemia was greater than that for macrovascular disease. Another meta-analysis of 4 randomized controlled trials of intensified glycemic control suggested that cancer risk was not reduced by improving glycemic control in type 2 diabetes and did not support the hypothesis that hyperglycemia is causally linked to increased cancer risk (29). Our study offers support for this conclusion and further indicates that lifestyle may play a more significant role in predicting the cancer risk of patients with diabetes.

Very few studies like the China Cardiometabolic Disease and Cancer Cohort study consider both lifestyle behaviors (smoking, physical activity, body mass index, and fruit and vegetable intake) and metabolic measures (total cholesterol, BP, and HbA1c) among diabetic patients (6). After a mean 3.8 years of follow-up of 25,860 diabetic patients, compared with patients with 1 ideal cardiovascular health metric or none, those with 5 or more were associated with 61% lower CVD risks. The present study further adds to the evidence that both the low-risk lifestyle and ideal level of BP and glycemic contribute to reduced risk of all-cause mortality and macrovascular and microvascular complications, but only the low-risk lifestyle contributes to the reduced risk of cancer mortality.

The evidence on the relative importance of glycemic and BP control for diabetes-related cardiovascular risk is inconclusive, with either one having been shown to be more important than the other (30, 31). One study involving 271,174 Swedish patients with type 2 diabetes compared the relative importance of various risk factors in predicting outcomes. The 2 strongest predictors were smoking and physical activity for the risk of death, and HbA1c and SBP for stroke and acute myocardial infarction (7). As far as we know, the present study is the first attempt to compare the relative importance of a combined low-risk lifestyle vs meeting BP and glycemic goals for preventing various diabetes-related outcomes. In this Chinese diabetes population, adopting a low-risk lifestyle was important in predicting all outcomes of interest except for microvascular complications. Ideal glycemic control was a predictor of the apparent greatest importance to microvascular complications and the low-risk lifestyle to cancer mortality. Ideal BP control showed mainly prognostic importance for macrovascular complications. The findings were generally robust across various subpopulations, with only a few exceptions. For example, the low-risk lifestyle had diminished importance for women, partially because of a significantly lower prevalence of smoking and excessive alcohol consumption among Chinese women. Such a variation suggests that the important factors in predicting outcomes may vary for different populations.

The strengths of this prospective study include long-term follow-up and a large number of outcome cases. Comprehensive information was collected on sociodemographic, lifestyle, and health factors that allowed control for confounding by other risk factors. Several limitations should also be considered. First, we only used information on lifestyle and health factors and disease treatment collected at baseline and did not consider their changes during follow-up. However, most participants did not change their lifestyle during a median interval of approximately 8 years. Second, measurement errors were inevitable for self-reported lifestyle factors. Third, we did not measure fasting blood glucose or HbA1c for all participants. The evaluation of glycemic control relied on RBG. Nevertheless, we have observed clear associations between RBG and outcomes of interest. Fourth, cholesterol control is important for cardiovascular health but was not included in the present study because lipid information was not available for all participants. Only the information on statin use was controlled for possible confounding. Fifth, information on diabetes-related microvascular complications was from hospital records, which may result in underestimation of these diseases, particularly for diseases with mild symptoms. Sixth, the nature of observational study precludes us from drawing a causal conclusion.

## Conclusions

In this Chinese population, there was a very low prevalence of diabetic patients who adhered to a low-risk lifestyle and had well-controlled BP and RBG. Having low-risk lifestyle and health factors were associated with reduced risk of all-cause and cancer mortality and macrovascular and microvascular complications among patients with diabetes. Assuming that the observed association is causal, the adoption of a low-risk lifestyle should be regarded as important as the achievement of ideal BP and glycemic goals in the prevention and management of diabetes-related health outcomes.

## Data Availability

Some or all data generated or analyzed during this study are included in this published article or in the data repositories listed in References. The access policy and procedures are available at www.ckbiobank.org. Details of how to access China Kadoorie Biobank data and details of the data release schedule are available from www.ckbiobank.org/site/Data+Access.

## Abbreviations

BP: blood pressure
CKB: China Kadoorie Biobank
CVD: cardiovascular disease
HbA1c: glycated hemoglobin
HR: hazard ratio
HS: hemorrhagic stroke
MCE: macrovascular complication
RBG: random blood glucose
SBP: systolic blood pressure
WHR: waist-to-hip ratio

## Notes

Members of the China Kadoorie Biobank collaborative group: International Steering Committee: Junshi Chen, Zhengming Chen (principal investigator [PI]), Robert Clarke, Rory Collins, Yu Guo, Liming Li (PI), Jun Lv, Richard Peto, Robin Walters. International Co-ordinating Centre, Oxford: Daniel Avery, Ruth Boxall, Derrick Bennett, Yumei Chang, Yiping Chen, Zhengming Chen, Robert Clarke, Huaidong Du, Simon Gilbert, Alex Hacker, Mike Hill, Michael Holmes, Andri Iona, Christiana Kartsonaki, Rene Kerosi, Garry Lancaster, Sarah Lewington, Kuang Lin, John McDonnell, Iona Millwood, Qunhua Nie, Jayakrishnan Radhakrishnan, Paul Ryder, Sam Sansome, Dan Schmidt, Paul Sherliker, Rajani Sohoni, Becky Stevens, Iain Turnbull, Robin Walters, Jenny Wang, Lin Wang, Neil Wright, Ling Yang, Xiaoming Yang. National Co-ordinating Centre, Beijing: Yu Guo, Xiao Han, Can Hou, Jun Lv, Pei, Chao Liu, Canqing Yu, Qingmei Xia. Ten regional coordinating Centers: Qingdao CDC: Zengchang Pang, Ruqin Gao, Shanpeng Li, Shaojie Wang, Yongmei Liu, Ranran Du, Yajing Zang, Liang Cheng, Xiaocao Tian, Hua Zhang, Yaoming Zhai, Feng Ning, Xiaohui Sun, Feifei Li. Licang CDC: Silu Lv, Junzheng Wang, Wei Hou. Heilongjiang Provincial CDC: Mingyuan Zou, Ge Jiang, Xue Zhou. Nangang CDC: Liqiu Yang, Hui He, Bo Yu, Yanjie Li, Qinai Xu, Quan Kang, Ziyan Guo. Hainan Provincial CDC: Dan Wang, Ximin Hu, Jinyan Chen, Yan Fu, Zhenwang Fu, Xiaohuan Wang. Meilan CDC: Min Weng, Zhendong Guo, Shukuan Wu, Yilei Li, Huimei Li, Zhifang Fu. Jiangsu Provincial CDC: Ming Wu, Yonglin Zhou, Jinyi Zhou, Ran Tao, Jie Yang, Jian Su. Suzhou CDC: Fang Liu, Jun Zhang, Yihe Hu, Yan Lu, Liangcai Ma, Aiyu Tang, Shuo Zhang, Jianrong Jin, Jingchao Liu. Guangxi Provincial CDC: Zhenzhu Tang, Naying Chen, Ying Huang. Liuzhou CDC: Mingqiang Li, Jinhuai Meng, Rong Pan, Qilian Jiang, Jian Lan,Yun Liu, Liuping Wei, Liyuan Zhou, Ningyu Chen Ping Wang, Fanwen Meng, Yulu Qin, Sisi Wang. Sichuan Provincial CDC: Xianping Wu, Ningmei Zhang, Xiaofang Chen, Weiwei Zhou. Pengzhou CDC: Guojin Luo, Jianguo Li, Xiaofang Chen, Xunfu Zhong, Jiaqiu Liu, Qiang Sun. Gansu Provincial CDC: Pengfei Ge, Xiaolan Ren, Caixia Dong. Maiji CDC: Hui Zhang, Enke Mao, Xiaoping Wang, Tao Wang, Xi zhang. Henan Provincial CDC: Ding Zhang, Gang Zhou, Shixian Feng, Liang Chang, Lei Fan. Huixian CDC: Yulian Gao, Tianyou He, Huarong Sun, Pan He, Chen Hu, Xukui Zhang, Huifang Wu. Zhejiang Provincial CDC: Min Yu, Ruying Hu, Hao Wang. Tongxiang CDC: Yijian Qian, Chunmei Wang, Kaixu Xie, Lingli Chen, Yidan Zhang, Dongxia Pan, Qijun Gu. Hunan Provincial CDC: Yuelong Huang, Biyun Chen, Li Yin, Huilin Liu, Zhongxi Fu, Qiaohua Xu. Liuyang CDC: Xin Xu, Hao Zhang, Huajun Long, Xianzhi Li, Libo Zhang, Zhe Qiu.

## References

[1] International Diabetes Federation. IDF Diabetes Atlas, 9th ed. 2019.

[2] Rao Kondapally Seshasai S , Kaptoge S, Thompson A, et al Diabetes mellitus, fasting glucose, and risk of cause-specific death. N Engl J Med. 2011;364(9):829-841. 21366474 10.1056/NEJMoa1008862 PMC4109980

[3] Harding JL , Pavkov ME, Magliano DJ, Shaw JE, Gregg EW. Global trends in diabetes complications: a review of current evidence. Diabetologia. 2019;62(1):3-16. 30171279 10.1007/s00125-018-4711-2

[4] Zhang Y , Pan XF, Chen J, et al Combined lifestyle factors and risk of incident type 2 diabetes and prognosis among individuals with type 2 diabetes: a systematic review and meta-analysis of prospective cohort studies. Diabetologia. 2020;63(1):21-33. 31482198 10.1007/s00125-019-04985-9

[5] Lv J , Yu C, Guo Y, et al Adherence to a healthy lifestyle and the risk of type 2 diabetes in Chinese adults. Int J Epidemiol. 2017;46(5):1410-1420. 28582543 10.1093/ije/dyx074 PMC5837408

[6] Wang T , Lu J, Su Q, et al Ideal cardiovascular health metrics and major cardiovascular events in patients with prediabetes and diabetes. JAMA Cardiol. 2019;4(9):874-883. 31365039 10.1001/jamacardio.2019.2499 PMC6669896

[7] Rawshani A , Rawshani A, Franzen S, et al Risk factors, mortality, and cardiovascular outcomes in patients with type 2 diabetes. N Engl J Med. 2018;379(7):633-644. 30110583 10.1056/NEJMoa1800256

[8] Chen Z , Lee L, Chen J, et al Cohort profile: the Kadoorie Study of Chronic Disease in China (KSCDC). Int J Epidemiol. 2005;34(6):1243-1249. 16131516 10.1093/ije/dyi174

[9] Chen Z , Chen J, Collins R, et al China Kadoorie Biobank of 0.5 million people: survey methods, baseline characteristics and long-term follow-up. Int J Epidemiol. 2011;40(6):1652-1666. 22158673 10.1093/ije/dyr120 PMC3235021

[10] Bragg F , Holmes MV, Iona A, et al Association between diabetes and cause-specific mortality in rural and urban areas of China. JAMA. 2017;317(3):280-289. 28114552 10.1001/jama.2016.19720 PMC6520233

[11] Sun Z. , Hu Y., Yu C., et al. (2022). Low-risk lifestyle and health factors and risk of mortality and vascular complications in Chinese patients with diabetes. *Zenodo*. Accessed March 20, 2022. 10.5281/zenodo.6371212

[12] Lloyd-Jones DM , Hong Y, Labarthe D, et al Defining and setting national goals for cardiovascular health promotion and disease reduction: the American Heart Association’s strategic Impact Goal through 2020 and beyond. Circulation. 2010;121(4):586-613. 20089546 10.1161/CIRCULATIONAHA.109.192703

[13] Arnett DK , Blumenthal RS, Albert MA, et al 2019 ACC/AHA Guideline on the Primary Prevention of Cardiovascular Disease: a report of the American College of Cardiology/American Heart Association Task Force on Clinical Practice Guidelines. J Am Coll Cardiol. 2019;74(10):e177-e232. 30894318 10.1016/j.jacc.2019.03.010 PMC7685565

[14] Chinese Diabetes Society. Guidelines for the prevention and control of type 2 diabetes in China (2020 Edition). Chinese Journal of Endocrinology and Metabolism. 2021;37(4):311-398.

[15] American Diabetes Association . 6. Glycemic targets: standards of medical care in diabetes-2019. Diabetes Care. 2019;42(Suppl 1):S61-S70. 30559232 10.2337/dc19-S006

[16] Harrell FE. Regression Modeling Strategies. Springer; 2015.

[17] Harrell FE . Package RMS. https://cran.r-project.org/web/packages/rms/rms.pdf.

[18] Long GH , Cooper AJ, Wareham NJ, Griffin SJ, Simmons RK. Healthy behavior change and cardiovascular outcomes in newly diagnosed type 2 diabetic patients: a cohort analysis of the ADDITION-Cambridge study. Diabetes Care. 2014;37(6):1712-1720. 24658389 10.2337/dc13-1731 PMC4170180

[19] Liu G , Li Y, Hu Y, et al Influence of lifestyle on incident cardiovascular disease and mortality in patients with diabetes mellitus. J Am Coll Cardiol. 2018;71(25):2867-2876. 29929608 10.1016/j.jacc.2018.04.027 PMC6052788

[20] Zhang Y , Tuomilehto J, Jousilahti P, Wang Y, Antikainen R, Hu G. Lifestyle factors on the risks of ischemic and hemorrhagic stroke. Arch Intern Med. 2011;171(20):1811-1818. 21911621 10.1001/archinternmed.2011.443

[21] Han Y , Hu Y, Yu C, et al Lifestyle, cardiometabolic disease, and multimorbidity in a prospective Chinese study. Eur Heart J. 2021;42(34):3374-3384. 34333624 10.1093/eurheartj/ehab413 PMC8423468

[22] Odegaard AO , Koh WP, Gross MD, Yuan JM, Pereira MA. Combined lifestyle factors and cardiovascular disease mortality in Chinese men and women: the Singapore Chinese health study. Circulation. 2011;124(25):2847-2854. 22104554 10.1161/CIRCULATIONAHA.111.048843 PMC3400937

[23] Lin CC , Li CI, Liu CS, et al Impact of lifestyle-related factors on all-cause and cause-specific mortality in patients with type 2 diabetes: the Taichung Diabetes Study. Diabetes Care. 2012;35(1):105-112. 22124717 10.2337/dc11-0930 PMC3241333

[24] Bonaccio M , Di Castelnuovo A, Costanzo S, et al Impact of combined healthy lifestyle factors on survival in an adult general population and in high-risk groups: prospective results from the Moli-sani Study. J Intern Med. 2019;286(2):207-220. 30993789 10.1111/joim.12907

[25] Diabetes Prevention Program Research Group. Long-term effects of lifestyle intervention or metformin on diabetes development and microvascular complications over 15-year follow-up: the Diabetes Prevention Program Outcomes Study. Lancet Diabetes Endocrinol. 2015;3(11):866-875. 26377054 10.1016/S2213-8587(15)00291-0 PMC4623946

[26] Gong Q , Zhang P, Wang J, et al Morbidity and mortality after lifestyle intervention for people with impaired glucose tolerance: 30-year results of the Da Qing Diabetes Prevention Outcome Study. Lancet Diabetes Endocrinol. 2019;7(6):452-461. 31036503 10.1016/S2213-8587(19)30093-2 PMC8172050

[27] Emdin CA , Rahimi K, Neal B, Callender T, Perkovic V, Patel A. Blood pressure lowering in type 2 diabetes: a systematic review and meta-analysis. JAMA. 2015;313(6):603-615. 25668264 10.1001/jama.2014.18574

[28] Stratton IM , Adler AI, Neil HA, et al Association of glycaemia with macrovascular and microvascular complications of type 2 diabetes (UKPDS 35): prospective observational study. BMJ. 2000;321(7258):405-412. 10938048 10.1136/bmj.321.7258.405 PMC27454

[29] Johnson JA , Bowker SL. Intensive glycaemic control and cancer risk in type 2 diabetes: a meta-analysis of major trials. Diabetologia. 2011;54(1):25-31. 20959956 10.1007/s00125-010-1933-3

[30] Afsharian S , Akbarpour S, Abdi H, et al Risk factors for cardiovascular disease and mortality events in adults with type 2 diabetes - a 10-year follow-up: Tehran Lipid and Glucose Study. Diabetes Metab Res Rev. 2016;32(6):596-606. 26787367 10.1002/dmrr.2776

[31] Wong ND , Patao C, Malik S, Iloeje U. Preventable coronary heart disease events from control of cardiovascular risk factors in US adults with diabetes (projections from utilizing the UKPDS risk engine). Am J Cardiol. 2014;113(8):1356-1361. 24581920 10.1016/j.amjcard.2013.12.042

